# Growing the Business of Whole Grain in the Australian Market: A 6-Year Impact Assessment

**DOI:** 10.3390/nu12020313

**Published:** 2020-01-24

**Authors:** Felicity Curtain, Alexandra Locke, Sara Grafenauer

**Affiliations:** 1 Grains & Legumes Nutrition Council, Mount Street, North Sydney, Sydney 2060, Australia; f.curtain@glnc.org.au (F.C.); a.locke@glnc.org.au (A.L.); 2 School of Medicine, University of Wollongong, Northfields Avenue, Wollongong 2522, Australia

**Keywords:** whole grain, food labelling, health claim, food regulation, public health

## Abstract

The Australia New Zealand Food Standards Code does not regulate on-pack claims describing the amount of whole grain in foods. In July 2013, The Grains & Legumes Nutrition Council^™^ (GLNC) established a voluntary Code of Practice for Whole Grain Ingredient Content Claims (the Code) providing guidance for whole grain claims, with cut-off values and suggested wording ≥8 g, ≥16 g, and ≥24 g per manufacturer serve (contains; high and very high in whole grain), based on a 48 g whole grain daily target intake. The aim of this impact assessment was to report the uptake of the Code by manufacturers, changes in numbers of whole grain products, and claims on-pack since 2013, including compliance. The impact assessment was undertaken in August 2019, comparing current registered manufacturers (“users”) and their products to the total number of products in the market deemed eligible for registration through GLNC product audits since 2013. Reporting included breakfast cereals, bread products, crispbreads, crackers, rice/corn cakes, rice, pasta, noodles, couscous, other grains (e.g., quinoa, buckwheat, freekeh), and grain-based muesli bars. As of 30 June 2019, there were 33 registered users and 531 registered products in Australia and New Zealand representing 43% of the eligible manufacturers and 65% of the eligible whole grain foods. Three-quarters (78% and 74%) of the eligible breakfast cereals and bread products were registered with the Code in 2019, followed by 62% of grain-based muesli bars. Only 39% of crispbread, crackers, rice/corn cakes, and rice, pasta, noodles, couscous, and other grains were registered. From 2013 there has been a 71% increase in the number of whole grain foods making claims, demonstrating strong uptake by industry, with clearer, more consistent, and compliant on-pack communication regarding whole grain content.

## 1. Introduction

Food labelling is considered an important policy tool in promoting healthier food choices, and includes nutrition content and health claims that draw attention to certain health benefits or nutritional characteristics of a product [1]. In Australia, such voluntary claims are regulated by Food Standards Australia New Zealand (FSANZ), who outline conditions that must be met in order to make claims [2]. While FSANZ provides a definition for whole grain—‘the intact grain or the dehulled, ground, milled, cracked or flaked grain where the constituents—endosperm, germ and bran—are present in such proportions that represent the typical ratio of those fractions occurring in the whole cereal, and includes wholemeal’ [3]—they do not regulate the use of on-pack whole grain claims, as with nutrients, such as dietary fibre, energy, vitamins, minerals, protein, carbohydrate, and fat [2].

In order to create a standard for whole grain labelling in Australia and New Zealand, in 2013, the Grains & Legumes Nutrition Council^™^ (GLNC) established a voluntary Code of Practice for Whole Grain Ingredient Content Claims (the Code) [4]. Registration with the Code is offered as a complimentary service to manufacturers of whole grain food products, based on the aim of establishing clear and consistent on-pack messaging around whole grain. Recommendations in the Code are set against GLNC’s established 48 g daily target intake (DTI) for whole grain, which was developed for adults and children aged 9 years and older, in consultation with an expert round table, and aligned with the target suggested in the American Dietary Guidelines [5,6]. GLNC also developed a separate DTI for children aged 4–8 years (32–40 g), and for toddlers aged 1–3 years (24 g) [4].

The Code also outlines a minimum whole grain content of 8 g per manufacturer serve to make a claim, based on the Australian Dietary Guidelines minimum suggestion of six serves of grain foods each day [7], combined with the 48 g DTI.

Suggested wording for claims is provided for:

- ‘DTI statements,’ (e.g., one (product serve descriptor) serve/s of (insert registered product name) contributes XX% of the 48 g whole grain daily target intake), and
- ‘Content claims,’ based on three levels of whole grain per manufacturer serve; ≥8 g (‘contains whole grain’), ≥16 g (‘high in whole grain’), and ≥24 g (‘very high in whole grain’), and/or factual statements about whole grain content (such as ‘67% whole grain’ or ‘22 g whole grain per serve’).

The Code’s development was deemed necessary based on the clear disconnect between advice to include whole grain foods, and current consumption levels. Recommendations to choose ‘mainly whole grain’ foods has featured consistently in Australian Dietary Guidelines since their inception in 1979 [8], and is similarly promoted in many other international guidelines, such as New Zealand [9], Canadian [10], Chinese [11], Danish [12], French [13], German [14], Singaporean [15], Swedish [16], Swiss [17], and the United Kingdom [18]. Despite this, The Lancet Global Burden of Disease study found that all 195 included countries fell severely short of optimal whole grain levels [19]. Pooled cross-section data of 7044 adults from a Western Australian (state-based) study from 1995, 1998, 2001, 2004, 2009, and 2012 provides data over time, just prior to the introduction of the Code. By 2012, the odds of consuming whole grain cereal foods increased since 2009 (OR = 1.27; 1.02–1.58 versus 1995 (*p* < 0.05) [20]. In 1995, 51.1% of participants consumed no whole grain foods, and this reduced to 37.2% in 2009 (*p* < 0.001). National data estimates whole grain at 21 g per day for adults, less than half of the 48 g DTI, and 30% consider themselves as ‘non-consumers’ of whole grain [21]. Interestingly, those consuming low or no whole grains still included similar amounts of grain-based foods but chose refined grain options instead of whole grain [21], with other research noting the main barriers to whole grain as negative sensory perception, lack of knowledge on how to find them, and lack of awareness of their health benefits [22,23]. In 2017, GLNC conducted a consumption study of 1200 participants matched with Australian Bureau Statistics demographic data. The sample was controlled for those consuming a gluten-free diet to ensure this population was not over-represented. Consumption of whole grains was determined at 26 g, a figure similar to the national data [24]. Modelling for the last dietary guidelines suggested a 160% increase in current wholegrain consumption and a 30% decrease in refined grain (cereal) food consumption was needed to meet the recommendations within the 2013 dietary guidelines [25].

This low level of consumption is concerning, as among dietary risk factors globally, diets low in whole grains were deemed the second leading risk factor for mortality (behind diets high in sodium), and the greatest risk factor for disability adjusted life years (DALYs), accounting for 82.5 million DALYs [19]. Extensive observational evidence supports the role of whole grains in promoting health, with consumption associated with reduced risk of cardiovascular disease, type 2 diabetes, overweight and obesity [26], all-cause mortality [27], and colorectal cancer [28]. Mechanisms for whole grains’ health benefits are thought to be a combination of their unique nutrition profile (providing dietary fibre, protein, B group vitamins, minerals, and phytochemicals) that may aid in reducing inflammation, lowering low density lipoprotein cholesterol, and maintaining blood glucose levels [26,27]. Whole grain consumers generally maintain a healthier overall diet, suggesting it may be a marker of healthier lifestyle habits [21,29].

Despite strong evidence for their health benefits, the general promotion through dietary guidelines, and low levels of consumption, the guidance and regulation of whole grain claims on food packaging in Australia remains voluntary. The aim of this study was (1) to evaluate the growth and engagement in whole grain products in the Australian market; (2) review the associated on-pack claims, including compliance; and (3) examine the impact of the Code since 2013.

## 2. Methods

In order to evaluate the growth and engagement in whole grain products in the Australian market, registered users of the Code and their products (≥8 g whole grain per serve) were calculated, and compared with product data collected on-shelf in Australian supermarkets. As GLNC’s grain food audit is restricted to products available in Australia, registered products available only in New Zealand were excluded from this research.

To assess on-pack whole grain claims, product data collected from December 2013–July 2014, (‘Audit 1’) and September 2017–30 June 2019 (‘Audit 2’) through GLNC’s established rolling audit of grain food were analysed. An audit is defined as a ‘systematic review or assessment of something’ [30]. This recognised process has been published previously [31,32,33,34], and was conducted in the four major supermarket chains (Aldi, Coles, Woolworths, IGA) in metropolitan Sydney, in order to capture the breadth of products available in Australia. Smartphones were used to record all on-pack information, including ingredients, nutrition information, nutrition and health claims, and any logos or endorsements, including the Health Star Rating. The categories included were breakfast cereals (including ready-to-eat cereals, breakfast biscuits, muesli, granola, clusters, hot cereals), bread, crispbreads, crackers, rice/corn cakes, pasta, rice, noodles, couscous, other grains (including quinoa, buckwheat), and grain-based muesli bars. Two categories of registered products (ready meals and grain-based snacks) could not be compared as audit data were not collected in the specified timeframe, so were excluded from the analysis. An additional internet search followed the in-store data collection, using key words related to each subcategory (such as ‘bread’, ‘pasta’, or specific brand names) to ensure all products were included. All data were transcribed into a Microsoft Excel spreadsheet, and checked by an independent reviewer prior to analysis to detect errors.

Data analysis specifically focused on whole grain information on food packaging. This included whole grain ingredients and their percentage, which were used to calculate the amount of whole grain in grams per serve, using percentage labelling as outlined by FSANZ [35], and any claims or wording that described whole grain content (both as described within the Code, and those outside the scope of the Code).

Current manufacturers utilising the Code, and their products, were compared with the total number of products collected through audits deemed eligible for registration (≥8 g whole grain per serve) to determine the portion of the market represented by the Code.

Price data was determined for products by conducting an Internet search through Coles and Woolworths online. This was used to compare whole grain and refined grain products to detect differences, with those unavailable online excluded from this comparison.

Additionally, changes in the Code registrations, whole grain claims, and whole grain content across categories were determined by comparing data collected from the same categories in audit 1 and audit 2. Only equivalent categories were compared, with products not collected at both time points removed from the data set.

### Statistics

Whole grain content data (grams per manufacturer serve) within each of the five grain food categories (and total data) were checked for normality using the Shapiro–Wilk test (IBM SPSS^®^, version 25.0, IBM Corp., Chicago, IL, USA) and the mean and standard deviation were presented. Independent samples t-tests were used to compare the change in the whole grain content between the two audit time periods and for the total whole grain captured through this audit process. T-tests were also used to compare the cost of refined versus whole grain food products recorded through GLNC audits and statistics were presented for total products, and between categories.

## 3. Results

As of June 2019, there were 664 registered whole grain products in the Australian market. After excluding 26 ready meals (e.g., grain-based salads, pasta, and rice-based dishes) and 44 grain-based snacks (e.g., popcorn, whole grain chips), which were not captured in GLNC’s product audit (as described in the methods section), 531 products remained (Table 1). The majority of these products were breakfast cereals (51%), breads (22%), and grain-based muesli bars (11%), with the remaining two categories (crispbreads, crackers, rice/corn cakes and pasta, rice, noodles, couscous, other grains) making up a smaller proportion.

Registered products were then compared with those captured through GLNC’s 2017–2019 product audits (audit 2; n = 1960). As shown in Table 1, nearly two-thirds (65%) of the total eligible whole grain products were registered with GLNC, with the greatest representation from the breakfast cereal (78% of registered products) and bread categories (74%). In total, 62% of grain-based muesli bars were registered, along with 39% of the remaining two categories.

At 30 June 2019, there were 33 registered manufacturers utilising the Code. A potential 45 additional manufacturers producing eligible whole grain products (≥8 g whole grain per serve) were identified through GLNC audits. Therefore, as of 30 June 2019, GLNC, through the Code, was engaged with 42% of the eligible manufactures in the Australian market who produce whole grain food products.

The number and type of whole grain claims were compared between categories, based on information gathered through GLNC’s 2017–2019 audit (Table 2). Overall, 29% of the products made a claim around whole grain: Breakfast cereals made up the greatest proportion of these (61%); followed by half of all grain-based muesli bars; a quarter (26%) of crispbreads, crackers, rice/corn cakes; 17% of bread products; and only 7% of pasta, rice, noodles, couscous, and other grains. Of the products making a claim, content claims were the most common (35% of claims overall), based on the Code’s minimum whole grain content levels (e.g., ≥8 g per serve = ‘contains whole grain’). Within categories, content claims were most prominent on breakfast cereals (72%), grain-based muesli bars (55%), and bread products (36%). Daily target intake (DTI) statements, such as ‘65% of the Whole Grain Daily Target’, (as outlined in the Code), made up almost two-thirds (35%) of claims overall, and were popular in bread products (82%), and grain-based muesli bars (39%). Few products were non-compliant with the guidelines outlined in the Code, aside from 12 bread products. Eleven of these displayed DTI claims that were in excess of their eligibility (e.g., stated 110% of the 48 g DTI was provided in one serve, where only 100% was provided). The remaining product, which was not registered with the Code, featured a claim that referred to the ‘RDI’ (recommended dietary intake) of whole grains, though this does feature within the Australian and New Zealand government nutrient reference values [36]. Conversely, claims outside the scope of the Code, which included non-evidence-based claims, like ‘Goodness of Whole Grains’, and ‘Packed with Whole Grains’, were found on 21% of the products but were most common on grain-based muesli bars and crispbreads, crackers, rice/corn cakes (both 31%), and on only 6% of the bread products.

Price data were collated for 61% of all previously described grain foods (n = 1203), and a comparison was made between whole grain and refined grain products, as presented in Table 3. Overall, there was a small but statistically significant price difference, with whole grain foods $0.77 more expensive than refined grain foods. Within categories, only whole grain pasta, rice, noodles, couscous, and other grains were significantly more expensive than refined grain (+$1.22). While there was no difference within the total bread category, when sliced loaf breads were analysed separately from the total category (excluding rolls, flatbreads, and fruit breads), refined sliced loaves were significantly more expensive then whole grain (+$0.46) (Table 3).

By comparing equivalent product data, collected 2013–2015 (‘audit 1’) through the same audit process as 2017–2019 (‘audit 2’), the impact of the Code was further determined (Table 4). Changes in the number of products registered with the Code, number of whole grain claims, mean whole grain content, and number of products not including the percentage of whole grain ingredients were analysed. Overall, a greater number of products were captured in audit 2 (1422 products compared with 1960 products), and an 11% increase in whole grain products compared to non-whole grain products. The number of products registered with the Code grew substantially overall and within each category, particularly breakfast cereals (1250% increase) and grain-based muesli bars (109% increase). There were new registrations in both crispbreads, crackers, and rice/corn cakes; and pasta, rice, noodles, couscous, and other grains, where previously GLNC had no registered products. The percentage of products making whole grain claims (including both those registered with the Code, and those not) grew by 71% in number, mainly attributed to crispbreads, crackers, and rice/corn cakes, which increased by 292%, and pasta, rice, noodles, couscous, and other grains, which had 280% more claims. Interestingly, whole grain claims in the bread category grew by only a modest 5%, and those on grain-based muesli bars decreased by 7%. Despite consistent growth in product registration with the Code, the mean whole grain content (g per serve) did not vary significantly overall (*p* = 0.069); however, there was a significant difference in the whole grain content between bars (*p* = 0.028) between the two audit periods, increasing from 13.4 ± 5 to 15.1 ± 5 g. Similarly, an additional 14% of products did not report the percentage of whole grain ingredients on-pack. Only grain-based muesli bars and breakfast cereals saw a decrease in this metric, dropping by 70% and 1.7%, respectively (Table 4).

## 4. Discussion

In the six years since its development, the Code has registered nearly two-thirds of the eligible whole grain products, the number of whole grain foods on-shelf has increased, and communication about whole grain on food packaging is more common. Few products were non-compliant with the Code (i.e., making a whole grain claim but not meeting the minimum 8 g requirement to do so). This opposes findings from a 2017 paper that found poor adherence with whole grain claims and the Code in grain foods, though this relied on 2015 data, and suggests manufacturers have made changes to align with the Code in the years since [37]. Nevertheless, an opportunity for continued engagement was evident by 21% of the products displaying claims outside the scope of the Code, such as ‘goodness of whole grains’, which are not evidence based. The number of whole grain products (compared with non-whole grain) increased by 11% between audit 1 and audit 2, though this is possibly underestimated, as products not stating the percentage of whole grain ingredients were considered a refined grain. However, as data from ready meals, grain-based snacks, flour, and toddler foods were not available at both data points for comparison, the entire category is now larger, and it is possible that new whole grain products may not have been captured. In fact, the marketing intelligence agency Mintel reported 1317 new product launches with whole grain claims in Australia between July 2013 and June 2019 [38], and although this includes deleted products, it suggests that an even greater number of products have entered the market than those captured through the GLNC supermarket audit process. Findings from the current study suggest new product development using refined grain foods surpassed those using whole grains, which aligns with Australian grain consumption preferences. Only around one third (34%) of grain foods eaten in the 2011–2012 National Nutrition and Physical Activity Survey were whole grain [39]. Similarly, the mean whole grain (g) content between audit 1 and audit 2 remained fairly constant, though at 29.5 g per serve (mean whole grain content), this equates to 61% of the 48 g whole grain DTI, demonstrating considerable amounts of whole grain in commonly consumed foods. This data supports the value in suggesting ‘swapping’ non-whole grain foods, such as bread, rice, and pasta, to whole grain options to improve dietary quality. This concept was demonstrated in a recent Irish study, where regression analysis found a 10 g daily increase of whole grain crackers or popcorn would lead to daily whole grain increases of 7.5 g, potentially 15% of the 48 g DTI [40].

Though the Code has led to more consistent and reliable whole grain labelling, a gap exists in consumer use and understanding of whole grain claims. Only one American study has reviewed dietitian understanding and promotion of the Whole Grain Stamp, a logo that draws similarities to the intent of the Code. Developed by the non-profit Whole Grains Council in 2005, the Whole Grain Stamp is an on-pack label that identifies products with 100% or 50% whole grains, with minimum levels of 16 and 8 g set for each. Around half (45%) of the 139 dietitians surveyed were familiar with, and likely to promote the system, with younger participants more likely to be aware than those 41 years or over [41]. More broadly, consumer perception of currently regulated nutrition claims (such as those indicating protein, sugar, fat, and dietary fibre content) is mixed, with some research supporting their role in promoting healthier food purchase, and others indicating the opposite based on lowered taste expectations [42,43,44]. When viewed in isolation, there is a criticism that health and nutrition content claims can lead to cognitive bias, or viewing a food as healthier based on a single nutritional aspect. Front-of-pack labelling (FOPL) systems that summarise a product’s nutrition information in an easily interpreted symbol, wording, or figure are thought to overcome the risk of cognitive bias, though few systems internationally consider whole grain content in their criteria [45].

Recognition of whole grains is currently a key area of consideration in a 5-year review of the Health Star Rating (HSR), the voluntary FOPL system in Australia and New Zealand [46]. The system ranks packaged foods on a scale between 0.5 (1/2) to 5 stars, with more stars indicating a healthier product, and scores foods based on their ‘negative nutrients’ (kilojoules, sodium, saturated fat, and total sugars), and ‘positive’ points, including fruit, vegetables, nuts, or legumes, as well as dietary fibre and protein [47]. The HSR does not directly reward grain foods for containing whole grain, and research has shown this translates to only minor score differences between refined and whole grain foods, such as white and brown rice, or pasta [48], but also snacks, such as muesli bars [34], failing to adequately communicate whole grain’s health benefits. This omission has been justified by the fact that some whole grain foods may achieve a higher score based on their dietary fibre content; however, fibre is known as a poor indicator of whole grain. In fact, the nutrition profiles of various whole grains vary significantly; the dietary fibre content of bulgur and barley is five times that of brown rice, and other nutrients like vitamin A and beta-carotene are found in corn but not in brown rice, oats, and sorghum [49]. Despite this, more than half of dietitians surveyed in an American study reported identifying and promoting whole grains by referring to their dietary fibre content [41], highlighting knowledge gaps even among nutrition professionals. The Key Hole label, the longest-standing FOPL system, developed in 1989 in Sweden, and now used widely across Nordic countries, is unique in considering whole grain in its system. The Key Hole aims to promote healthier choices within categories based on requirements, such as less and healthier fats, less sugars, less salt, and more dietary fibre and whole grain [50]. Similarly, the Singapore Healthier Choices symbol, introduced in 1998, awards a symbol to ‘healthier’ foods within subcategories, accompanied by text, such as ‘higher in whole grains’, or ‘lower in sodium’ [51], with thorough criteria for foods to ensure accurate promotion of whole grain foods.

Although whole grain consumption in Australia is less than half of the DTI, it is clear that food labelling and FOPL systems cannot work in isolation to improve food choice. A systematic review of public health interventions aimed at increasing whole grain found successful programs were based on multiple stakeholders, outlined target intakes in dietary guidelines, and codes of practice for labelling whole grain foods [52]. Specifically, a collaborative multi-level approach was recommended, involving all components of the food system, such as academia, government, food industry, advocacy groups, product developers, and consumers. The Danish are one of the few nations to have substantially increased whole grain consumption in recent years, based on their successful Danish Wholegrain Public Private Partnership (PPP) [53]. Established in 2008, the PPP engaged 31 partners across areas, such as food regulation, non-government organisations, and food industry. Over a decade, the PPP saw national whole grain consumption increase by 75%, from 36 to 63 g per day/10 MJ. Those meeting the Danish recommended 75 g of whole grain per day also rose from 6% to 30% [53]. In order to achieve increases in whole grain consumption in Australia, a similar collaborative approach should be taken, though this relies upon support from current initiatives, such as the HSR.

The perception that whole grains are more expensive then refined grains is a well-established barrier to consumption [23,54], and though this was the case for the category overall, the difference was minor (less than $1AUD), and within categories related only to pasta, rice, noodles, couscous, and other grains. This may be explained by the heterogeneous nature of the category, including basic items, such as rice, as well as more niche ‘ancient grains’, like quinoa and buckwheat, often positioned as premium ingredients [55]. Interestingly, a Canadian study found breads with whole grain claims were likely to cost more than those without [56], opposing the findings of this study, in which white loaves were priced higher than whole grain. This shows both the potential for manufacturers to offer whole grain foods as a premium product, and also confirms price is not necessarily a true barrier when choosing between whole grain and refined grain foods. Small price differences could also be considered minor when compared to the potential savings in long-term healthcare expenditure and productivity. Considering the weight of evidence from the Global Burden of Disease studies, a cost-of-illness analysis would be useful to assess the potential savings associated with a lower prevalence of cardiovascular disease and type 2 diabetes resulting from the exchange of whole grain food items for refined grains. Modelling has been undertaken based on the Australian diet for three levels of increased dietary fibre intake from cereal fibre based on a 10% increase in total dietary fibre: An increase to the adequate intake (25 g for women and 30 g for men) and an increase to the suggested dietary target (25 g for women and 38 g for men) [57]. Based on the three levels, a range in the total combined annual healthcare expenditure and productivity cost savings was calculated from AUD$17.8 million to $1.6 billion for cardiovascular disease and from AUD$18.2 million to $1.7 billion for type 2 diabetes mellitus [58]. The authors found that the benefits were greater for adults of lower socioeconomic status and those with lower dietary fibre intakes, and a similar finding could be expected for whole grain foods. However, rather than adding whole grain food products, the emphasis would be on swapping as opposed to increasing, an important point considering the levels of obesity. This type of study is needed to highlight the substantial healthcare and productivity cost savings that could be realised through nutrition initiatives, particularly to gain government support.

For the food industry, new product development and whole grain on-pack claims are growing ahead of those focused on fibre, presenting a distinct opportunity. Mintel data shows 60% more new claims for whole grain (n = 27,871) compared with dietary fibre (n = 17,351) globally in the period from mid-2013 to mid-2019 [38]. This is particularly the case within food categories, such as pasta and noodles, which have few whole grain options widely available in Australia. Changing consumer demand has seen global interest rise in healthier formulations in these categories, using traditional whole grains like wheat, but also wild rice, millet, and quinoa [59]. Innovation using whole grain ingredients may also support the building focus on sustainable dietary patterns, such as targets described in the recently published EAT Lancet report. With food a critical element for both human and planetary health, plant-based foods, such as whole grains, present a unique opportunity to appeal to this important and topical issue [60].

Strengths of this study include its comprehensive nature, with data collected over two time frames, allowing for a comparison of changes. However, there were some limitations. While all efforts were made to capture the grain foods category in its entirety, there may be differences between geographic areas. Additionally, reported ingredients and nutrition information was relied upon, with no validation or independent nutrition analysis conducted, so some nutrients (including whole grain) were unable to be compared. Finally, product cost data for whole grain versus refined grain foods were only obtained for products available for Coles and Woolworths online at the time of analysis, meaning 40% of the products could not be compared for this metric.

## 5. Conclusions

As a result of the Code’s development in 2013, whole grain labelling in the Australian food supply has increased by 70%, enabling consumers to make informed evidence-based decisions when choosing grain-based foods at the supermarket shelf. The Code has achieved strong growth and engagement, representing almost two-thirds (65%) of all eligible whole grain foods. Improved labelling is a key aspect of encouraging healthier food choice, though a collaborative approach is required to address other barriers to whole grain consumption. Future considerations should include stronger quantified recommendations in dietary guidelines and food selection guides; promotion through other government-supported initiatives, such as the HSR; and health promotion campaigns, coupled with food industry innovation to create consumer-accepted whole grain products. Simple ‘swaps’ of refined grain for whole grain choices should be the basis for messaging as this would easily meet the 48 g DTI.

## Figures and Tables

**Table 1:** Grain food categories and registered products as of June 2019.

Total Products in Grain Food Categories	Number of Registered Products ^1^	Number of Eligible Unregistered Products ^2^	Estimated Code Uptake (%)
Breakfast cereals (n = 441)	243	65	78
Bread products (n = 456)	135	47	74
Crispbreads, crackers, rice/corn cakes (n = 363)	29	44	39
Pasta, rice, noodles, couscous, other grains (n = 535)	55	85	39
Grain-based muesli bars (n = 165)	69	41	62
Total (n = 1960)	531	285	65

^1^ Sourced from GLNC’s Registered Product Database ^2^ Sourced from GLNC’s 2017–2019 Grain Food Product Audit, defined as products that had ≥8 g whole grain/serve. Excludes products where whole grain content could not be assessed.

**Table 2:** Whole grain claims on-pack *.

Grain Food Categories	Any Whole Grain Claim (% of Total Products)	Daily Target Intake (DTI) Statement ^1^ (% of Products Making a Claim)	Content Claim (% of Products Making a Claim) ^2^	Claim Outside the Scope of the Code (% of Products Making a Claim) ^3^
Breakfast cereals (n = 441)	61% (n = 271)	25% (n = 69)	72% (n = 197)	18% (n = 49)
Bread products (n = 456)	17% (n = 82)	82% (n = 68)	36% (n = 30)	6% (n = 5)
Crispbreads, crackers, rice/corn cakes (n = 363)	26% (n = 98)	24% (n = 24)	25% (n = 25)	31% (n = 31)
Pasta, rice, noodles, couscous, other grains (n = 535)	7% (n = 38)	28% (n = 11)	26% (n = 10)	26% (n = 10)
Grain-based muesli bars (n = 165)	50% (n = 83)	39% (n = 33)	55% (n = 46)	31% (n = 26)
Total (n = 1960)	29% (n = 572)	35% (n = 205)	53% (n = 308)	21% (n = 121)

* Sourced from GLNC’s 2017–2019 Grain Food Product. ^1^ Based on contribution to 48 g whole grain DTI. ^2^ Based on level of whole grain. ^3^ Any whole grain claim not related to guidance set out in the Code.

**Table 3:** Price comparison of whole grain (WG) and refined grain (RG) foods.

Grain Food Categories	WG/RG	Price ($AUD)	*p* Value
Breakfast cereals (n = 266)	WG: n = 195	$5.34 ± 1.6	0.533
	RG: n = 71	$5.49 ± 2.1	
Bread products (n = 365)	WG: n = 111	$3.92 ± 1.5	0.448
	RG: n = 254	$4.06 ± 1.8	
Sliced loaf breads (n = 161) *	WG: n = 69	$4.01 ± 1.1	0.036
	RG: n = 92	$4.47 ± 1.6	
Crispbreads, crackers, rice/corn cakes (n = 203)	WG: n = 53	$2.84 ± 1.0	0.149
	RG: n = 150	$3.11 ± 1.5	
Pasta, rice, noodles, couscous, other grains (n = 283)	WG: n = 49	$4.33 ± 3.2	0.009
	RG: n = 234	$3.11 ± 2.9	
Grain-based muesli bars (n = 86)	WG: n = 55	$4.31 ± 1.5	0.566
	RG: n = 31	$4.48 ± 1.2	
Total (n = 1203) **	WG: n = 463	$4.49 ± 1.9	<0.001
	RG: n = 740	$3.72 ± 2.3	

* Sliced loaves were analysed in addition to total bread products. ** Based on products with prices available on-line.

**Table 4:** Changes in whole grain products, registered product claims, mean whole grain content, and proportion not reporting whole grain content 2013–2019, based on Grains & Legumes Nutrition Council audit data.

Category	Breakfast Cereals			Bread Products			Crispbreads, Crackers, Rice/Corn Cakes			Pasta, Rice, Noodles, Couscous, Other Grains			Grain-Based Muesli Bars			Total		
	Audit 1	Audit 2	% Change	Audit 1	Audit 2	% Change	Audit 1	Audit 2	% Change	Audit 1	Audit 2	% Change	Audit 1	Audit 2	% Change	Audit 1	Audit 2	% Change
Whole grain products vs. total category	171/274 *	308/441	80	135/468	130/436	−5	39/79	79/363	103	75/415	104/535	39	67/186	109/165	63	487/1442	730/1960	39
% Whole grain products	62	69	7	28	29	1	49	76	27	18	19	1	36	66	30	33	37	11
Registered with the Code (n=)	18	243	1250	69	135	95	0	29	NA	0	55	NA	33	69	109	120	531	342
Whole grain claims (n=) **	130	271	108	78	82	5	25	98	292	10	38	280	90	83	-7	333	572	71
Mean whole grain (±SD) g per serve	27.7 ± 9.5	27.6 ± 10.7	−0.3	29.2 ± 15.7	27.6 ± 14.5	−5.4	19.5 ± 5.9	17.9 ± 6.7	−8.2	68.3 ± 31.5	61.9 ± 24.4	−9.3	13.4 ± 5	15.1 ± 5	12.6	31.8 ± 22.9	29.5 ± 19.3	−7.2
Not reporting whole grain content (n=)	57	56	−1.7	62	78	25	9	40	344	2	12	500	44	13	−70	174	199	14

* Eligible whole grain products (≥8 g whole grain per serve)/total number of products captured ** Percentage of total products within audit category.

## References

[1] Wellard-Cole L., Watson W.L., Hughes C., Chapman K. (2019). How effective is food industry self-substantiation of food–health relationships underpinning health claims on food labels in Australia?. Public Health Nutr..

[2] Food Standards Australia New Zealand Australia New Zealand Food Standards Code—Standard 1.2.7—Nutrition, Health and Related Claims. www.comlaw.gov.au/Series/F2013L00054.

[3] Food Standards Australia New Zealand Wholegrain Food. http://www.foodstandards.gov.au/consumer/nutrition/wholegrain/Pages/default.aspx.

[4] GLNC Code of Practice for Whole Grain Ingredient Content Claims. http://www.glnc.org.au/codeofpractice/.

[5] Griffiths T. (2006). Developing a target for daily wholegrain intake for Australians. Food Aust..

[6] Griffiths T. (2007). Towards an Australian ‘daily target intake’ for wholegrains. Food Aust..

[7] National Health and Medical Research Council Grain (Cereal) Foods, Mostly Wholegrain and/or High Cereal Fibre Varieties. https://www.eatforhealth.gov.au/food-essentials/five-food-groups/grain-cereal-foods-mostly-wholegrain-and-or-high-cereal-fibre.

[8] Department of Community Services and Health (1987). Towards Better Nutrition for Australians. Report of the Nutrition Taskforce of the Better Health Commission.

[9] New Zealand Ministry of Health (2015). Eating and Activity Guidelines for New Zealand Adults.

[10] Health Canada (2019). Canada’s Dietary Guidelines.

[11] Wang S., Lay S., Yu H., Shen S. (2016). Dietary Guidelines for Chinese Residents (2016): Comments and comparisons. J. Zhejiang Univ..

[12] The Danish Veterinary and Food Administration Choose Whole Grains. https://altomkost.dk/raad-og-anbefalinger/de-officielle-kostraad/vaelg-fuldkorn/.

[13] Mangerbouge Programme National Nutrition Sante Starches. http://www.mangerbouger.fr/Les-9-reperes/Les-9-reperes-a-la-loupe/Les-feculents.

[14] German Nutrition Society Whole Grain. https://www.dge.de/ernaehrungspraxis/vollwertige-ernaehrung/10-regeln-der-dge/.

[15] Ministry of Health Singapore The Dietary Guidelines for Adult Singaporeans. https://www.healthhub.sg/live-healthy/15/dietary_guidelines_adults.

[16] Konde A.B., Njerselius R., Haglund L., Jansson A., Pearson M., Färnstrand J.S., Johansson A. Swedish Dietary Guidelines—Risk and Benefit Management Report. http://www.livsmedelsverket.se/globalassets/rapporter/2015/rapp-hanteringsrapport-engelska-omslag--inlaga--bilagor-eng-version.pdf.

[17] Food and Agricultural Organisation of the United Nations Food-Based Dietary Guidelines—Switzerland. http://www.fao.org/nutrition/education/food-dietary-guidelines/regions/countries/switzerland/en/.

[18] NHS Starchy Foods and Carbohydrates. https://www.nhs.uk/live-well/eat-well/starchy-foods-and-carbohydrates/.

[19] GBD 2017 Risk Factor Collaborators (2018). Global, regional, and national comparative risk assessment of 84 behavioural, environmental and occupational, and metabolic risks or clusters of risks for 195 countries and territories, 1990–2017: A systematic analysis for the Global Burden of Disease Study 2017. Lancet.

[20] Pollard C.M., Pulker C.E., Meng X., Scott J.A., Denham F.C., Solah V.A., Kerr D.A. (2017). Consumer attitudes and misperceptions associated with trends in self-reported cereal foods consumption: Cross-sectional study of Western Australian adults, 1995 to 2012. BMC Public Health.

[21] Galea L., Beck E., Probst Y., Cashman C. (2017). Whole grain intake of Australians estimated from a cross-sectional analysis of dietary intake data from the 2011-13 Australian Health Survey. Public Health Nutr..

[22] Kamar M., Evans C., Hugh-Jones S. (2016). Factors influencing adolescent whole grain intake: A theory-based qualitative study. Appetite.

[23] McMackin E., Dean M., Woodside J., McKinley M.C. (2013). Whole grains and health: Attitudes to whole grains against a prevailing background of increased marketing and promotion. Public Health Nutr..

[24] Suthers R. (2017). Whole Grain Consumption in Australia: Intake, demographics, barriers and facilitators. Thesis to Satisfy the Requirements for MSc (Nutrition).

[25] NHMRC (2011). A Modelling System to Inform the Revision of the Australian Guide to Healthy Eating.

[26] Ferruzzi M.G., Jonnalagadda S.S., Liu S., Marquart L., McKeown N., Reicks M., Riccardi G., Seal C., Slavin J., Thielecke F. (2014). Developing a Standard Definition of Whole-Grain Foods for Dietary Recommendations: Summary Report of a Multidisciplinary Expert Roundtable Discussion. Adv. Nutr. Int. Rev. J..

[27] Aune D., Keum N., Giovannucci E., Fadnes L.T., Boffetta P., Greenwood D.C., Tonstad S., Vatten L.J., Riboli E., Norat T. (2016). Whole grain consumption and risk of cardiovascular disease, cancer, and all cause and cause specific mortality: Systematic review and dose-response meta-analysis of prospective studies. Br. Med J..

[28] World Cancer Research Fund/American Institute for Cancer Research Wholegrains, Vegetables & Fruit. https://www.wcrf.org/dietandcancer/exposures/wholegrains-veg-fruit.

[29] Ruggieo E., Bonaccio M., Castelnuovo A.D., Bonanni A., Constanzo S., Persichillo M., Bracone F., Cerletti C., Donati M.B., Gaetano G.D. (2019). Consumption of whole grain food and its determinants in a general Italian population: Results from the INHES study. Nutr. Metab. Cardiovasc. Dis..

[30] Lexico Definition of Audit in English. https://www.lexico.com/en/definition/audit.

[31] Dunford E., Webster J., Metzler A., Czernichow S., Ni Mhurchu C., Wolmarans P., Snowdon W., L’Abbe M., Li N., Maulik P.K. (2012). International collaborative project to compare and monitor the nutritional composition of processed foods. Eur. J. Prev. Cardiol..

[32] Grafenauer S., Curtain F. (2018). An Audit of Australian Bread with a Focus on Loaf Breads and Whole Grain. Nutrients.

[33] Curtain F., Grafenauer S.J. (2019). Health Star Rating in Grain Foods—Does It Adequately Differentiate Refined and Whole Grain Foods?. Nutrients.

[34] Curtain F., Grafenauer S. (2019). Comprehensive Nutrition Review of Grain-Based Muesli Bars in Australia: An Audit of Supermarket Products. Foods.

[35] Food Standards Australia New Zealand Percentage Labelling of Food User Guide to Standard 1.2.10—Characterising Ingredients and Components of Food. https://www.foodstandards.gov.au/code/userguide/Documents/Guide%20to%20Standard%201.2.10%20-%20Percentage%20Labelling%20of%20Food.pdf.

[36] National Health and Medical Research Council Nutrient Reference Values for Australia & New Zealand. https://www.nrv.gov.au/nutrients.

[37] Pulker C., Trapp G., Scott J., Pollard C. (2018). Alignment of Supermarket Own Brand Foods’ Front-of-Pack Nutrition Labelling with Measures of Nutritional Quality: An Australian Perspective. Nutrients.

[38] Mintel (2019). Global New Product Database.

[39] Australian Bureau of Statistics No. 4364.0.55.012—Australian Health Survey: Consumption of Food Groups from the Australian Dietary Guidelines, 2011–2012. https://www.abs.gov.au/ausstats/abs@.nsf/Lookup/by%20Subject/4364.0.55.012~2011-12~Main%20Features~Grain%20(cereals)~10008.

[40] O’Donovan C.B., Devlin N.F., Buffini M., Walton J., Flynn A., Gibney M.J., Nugen A.P. (2018). Whole grain intakes in Irish adults: Findings from the National Adults Nutrition Survey (NANS). Eur. J. Nutr..

[41] Edwards A., Minihan K., Shenton S., Washington J., Edelstein S. (2008). Awareness of New Promotional Tools for Whole Grains Among Dietitians. Top. Clin. Nutr..

[42] Talati Z., Norman R., Kelly B., Dixon H., Neal B., Miller C., Pettigrew S. (2017). A randomized trial assessing the effects of health claims on choice of foods in the presence of front-of-pack labels. Am. J. Clin. Nutr..

[43] Kaur A., Scarborough P., Raynor M. (2017). A systematic review, and meta-analyses, of the impact of health-related claims on dietary choices. Int. J. Behav. Nutr. Phys. Act..

[44] Steinhauser J., Janssen M., Hamm U. (2019). Consumers’ purchase decisions for products with nutrition and health claims: What role do product category and gaze duration on claims play?. Appetite.

[45] Zenobia T., Simone P., Helen D., Bruce N., Kylie B., Hughes C. (2016). Do Health Claims and Front-of-Pack Labels Lead to a Positivity Bias in Unhealthy Foods?. Nutrients.

[46] Commonwealth of Australia, About Health Star Ratings. http://healthstarrating.gov.au/internet/healthstarrating/publishing.nsf/Content/About-health-stars.

[47] Jones A., Shahid M., Neal B. (2018). Uptake of Australia’s Health Star Rating System. Nutrients.

[48] MPconsulting Health Star Rating System Five Year Review Report. http://www.healthstarrating.gov.au/internet/healthstarrating/publishing.nsf/Content/D1562AA78A574853CA2581BD00828751/$File/Health-Star-Rating-System-Five-Year-Review-Report.pdf.

[49] De Moura F.F., Lewis K.D., Falk M.C. (2009). Applying the FDA Definition of Whole Grains to the Evidence for Cardiovascular Disease Health Claims. J. Nutr..

[50] van der Bend D., Lissner L. (2019). Differences and Similarities between Front-of-Pack Nutrition Labels in Europe: A Comparison of Functional and Visual Aspects. Nutrients.

[51] Singapore Health Promotion Board Healthier Choice Symbol Nutrient Guidelines. https://www.hpb.gov.sg/docs/default-source/default-document-library/hcs-guidelines-(january-2018)9ab599f6468366dea7adff00000d8c5a.pdf?sfvrsn=2d36ff72_0.

[52] Suthers R., Broom M., Beck E.J. (2018). Key Characteristics of Public Health Interventions Aimed at Increasing Whole Grain Intake: A Systematic Review. J. Nutr. Educ. Behav..

[53] Greve C., Iben Neess R. (2014). The Evolution of the Whole Grain Partnership in Denmark.

[54] Neo J., Brownlee I. (2017). Wholegrain Food Acceptance in Young Singaporean Adults. Nutrients.

[55] Vara V. Why We’re Willing to Pay More for Cereals with Ancient Grains. https://www.newyorker.com/business/currency/well-pay-ancient-grains-cheerios.

[56] Sumanac D., Mendelson R., Tarasuk V. (2013). Marketing whole grain breads in Canada via food labels. Appetite.

[57] National Health and Medical Research Council, Australian Government Department of Health and Ageing, New Zealand Ministry of Health Nutrient Reference Values for Australia and New Zealand. https://www.nrv.gov.au/chronic-disease/summary.

[58] Fayet-Moore F., Cassettari T., Tuck K., McConnell A., Petocz P. (2018). Dietary Fibre Intake in Australia. Paper I: Associations with Demographic, Socio-Economic, and Anthropometric Factors. Nutrients.

[59] Walji A. (2018). A Year of Innovation in Pasta, Rice & Noodles, 2018.

[60] Willett W. (2019). Food in the Anthropocene: The EAT-Lancet Commission on healthy diets from sustainable food systems. Lancet Glob. Health.

